# Middlesex District Councils' Association

**Published:** 1906-12-01

**Authors:** 


### MIDDLESEX DISTRICT COUNCILS' ASSOCIATION.

### THE PROPOSED SANATORIUM FOR TUBERCULOSIS.

On Monday a conference of Middlesex Councils was held at the Guildhall, Westminster, to consider a proposal of the Middlesex District Councils' Association that the local authorities in the county should combine for the purpose of providing hospital accommodation for persons suffering from consumption.

Mr. A. W. W. King (Acton), the Chairman of the Association, presided over a representative meeting, which was largely attended, twelve Councils sending representatives.

Mr. King having introduced the subject, Colonel Gerard Clark, the Hon. Secretary of the Executive Committee of the proposed Middlesex Open-Air Sanatorium, then gave an interesting address.

It was decided to form a sub-committee, consisting of Messrs. E. G. Cole (Wood Green), J. J. Bonnett (Heston and Isleworth), McCullum (Uxbridge), Henderson and Lascelles (Harrow), and Butler (Willesden), to go into the matter thoroughly, to obtain information as to the action (if any) taken by various districts, to communicate with the Mount Vernon Hospital authorities, and prepare and circulate full reports to be considered at an adjourned meeting of the Association.

